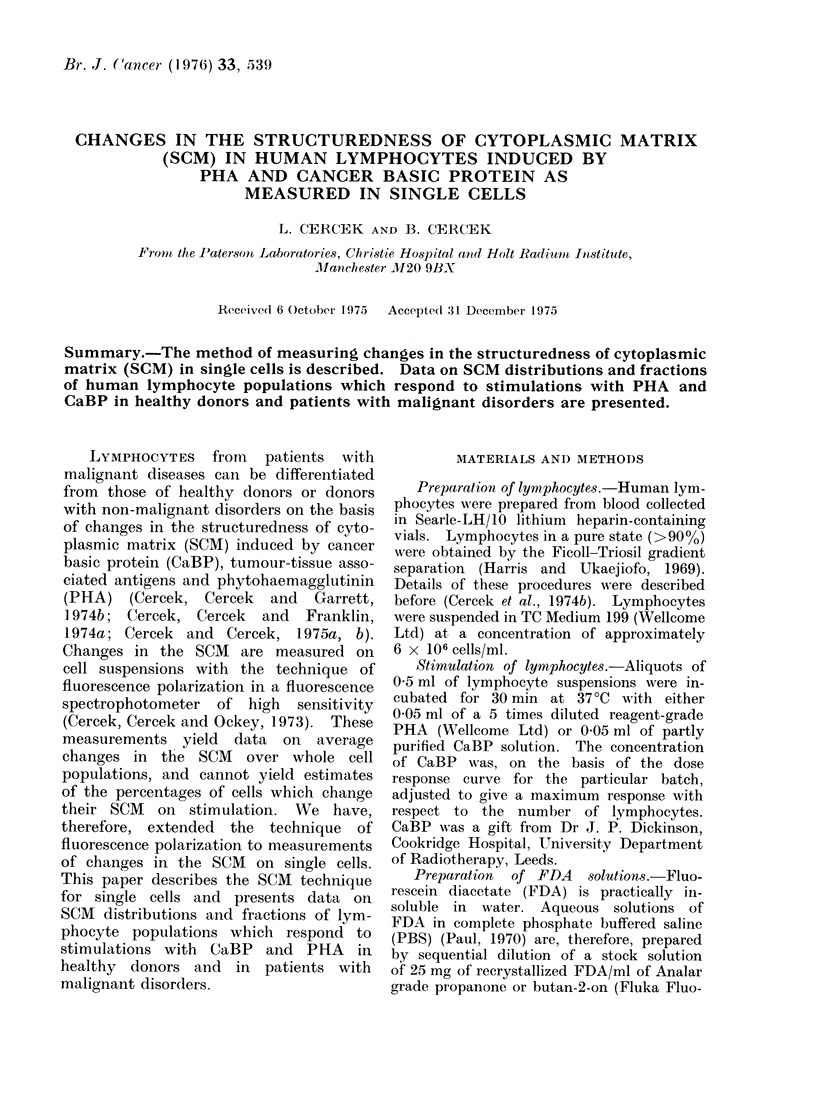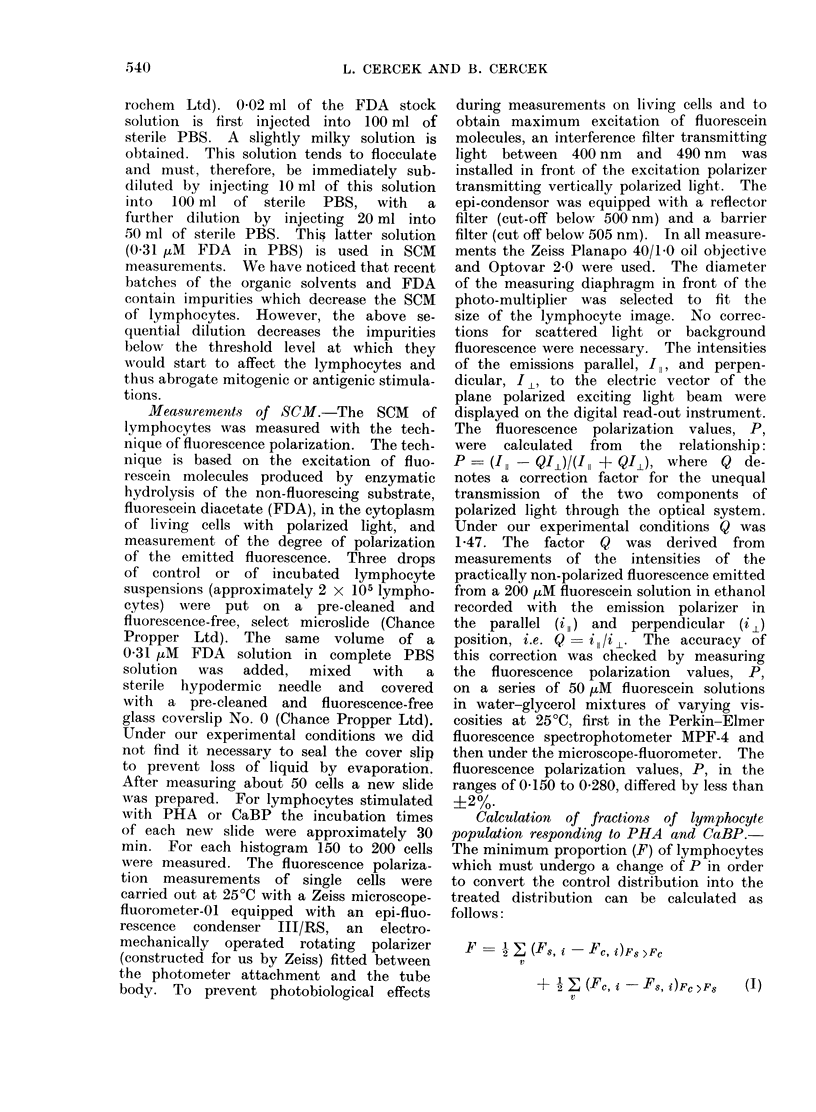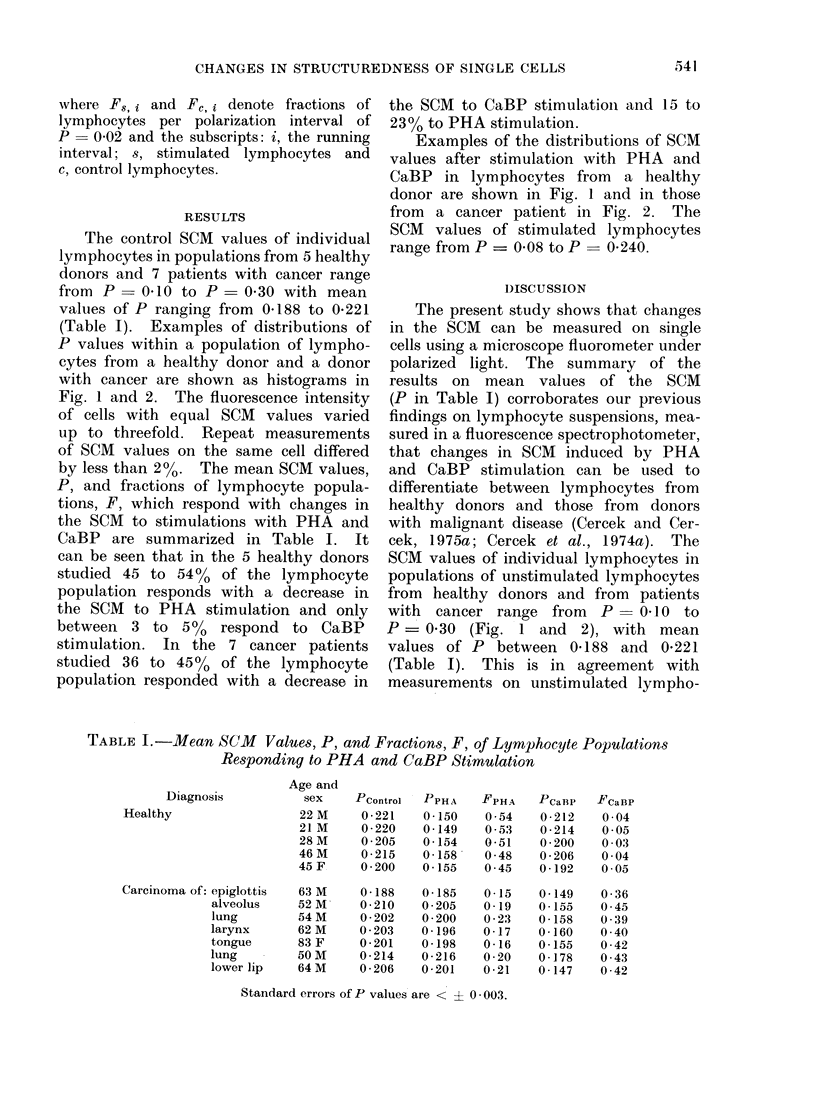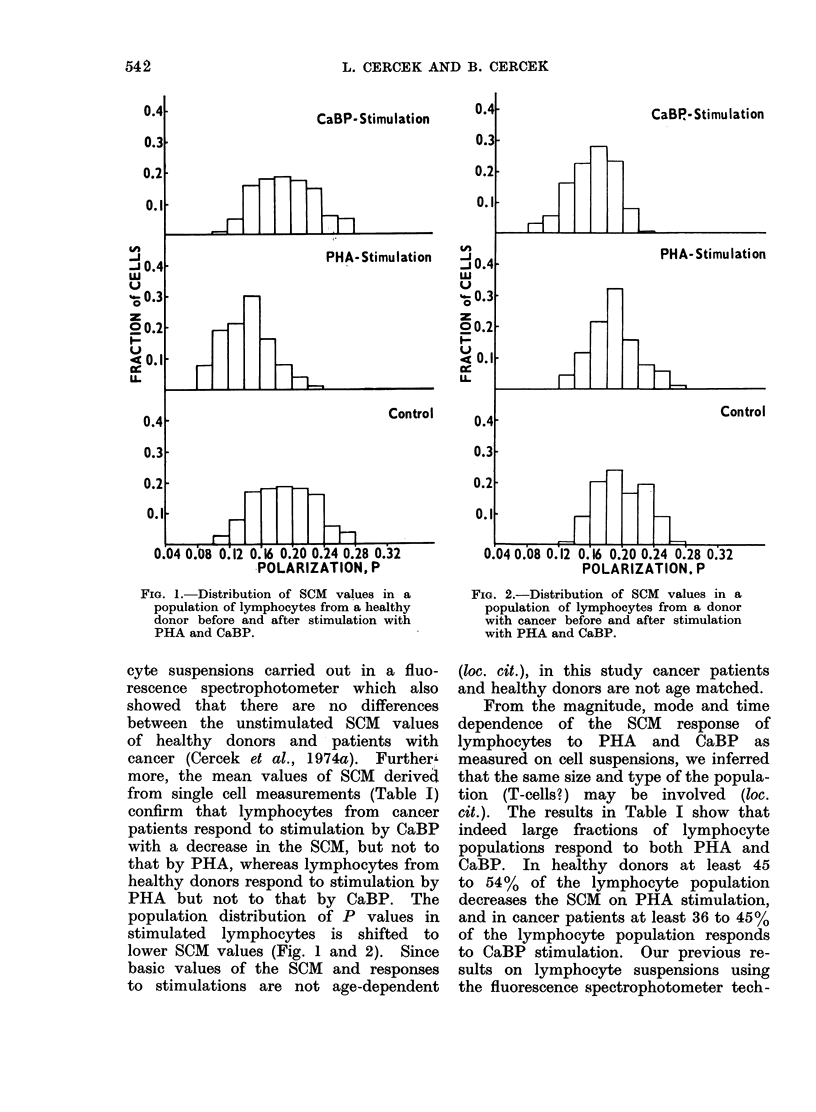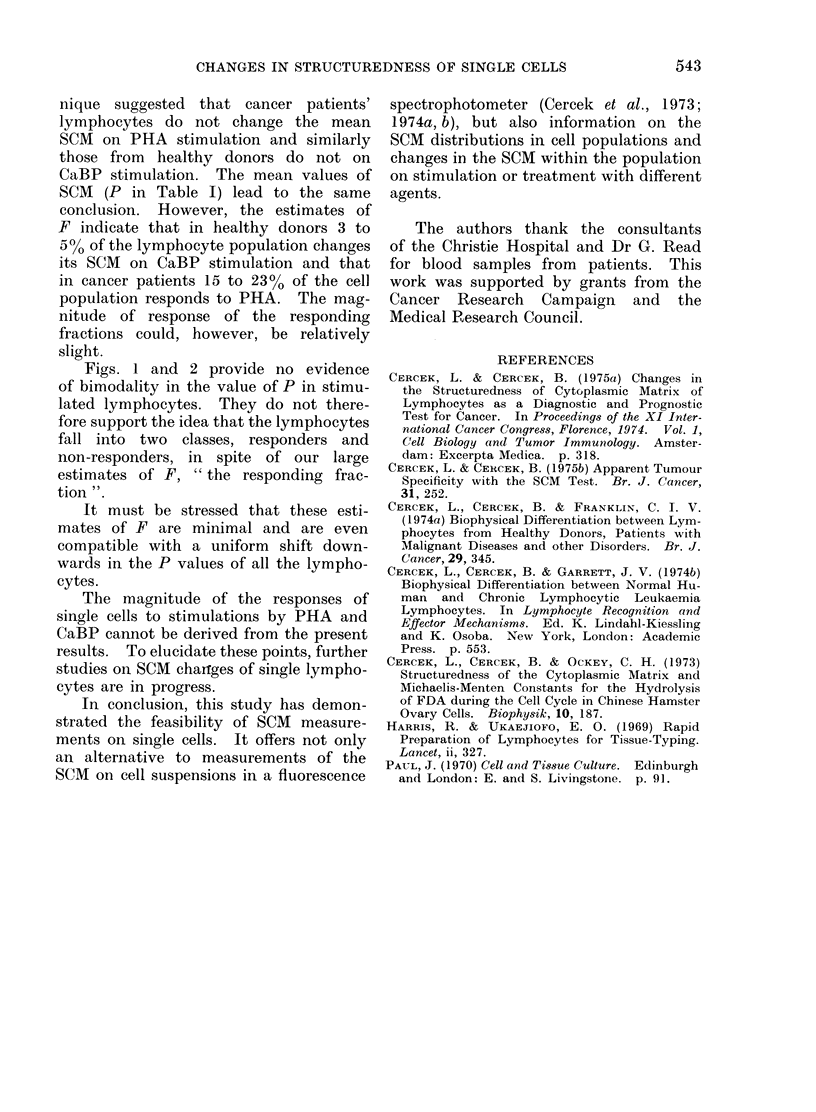# Changes in the structuredness of cytoplasmic matrix (SCM) in human lymphocytes induced by PHA and cancer basic protein as measured in single cells.

**DOI:** 10.1038/bjc.1976.85

**Published:** 1976-05

**Authors:** L. Cercek, B. Cercek

## Abstract

The method of measuring changes in the structuredness of cytoplasmic matrix (SCM) in single cells is described. Data on SCM distributions and fractions of human lymphocyte populations which respond to stimulations with PHA and CaBP in healthy donors and patients with malignant disorders are presented.


Br. J. Cancer (1976) 33, 539

### CHANGES IN THE STRUCTUREDNESS OF CYTOPLASMIC MATRIX (SCM) IN HUMAN LYMPHOCYTES INDUCED BY PHA AND CANCER BASIC PROTEIN AS MEASURED IN SINGLE CELLS

L. CERCEK AND B. CERCEK

*From the Paterson Laboratories, Christie Hospital and Holt Radium Institute, Manchester M20 9BX*

Received 6 October 1975 Accepted 31 December 1975

**Summary.—The method of measuring changes in the structuredness of cytoplasmic matrix (SCM) in single cells is described. Data on SCM distributions and fractions of human lymphocyte populations which respond to stimulations with PHA and CaBP in healthy donors and patients with malignant disorders are presented.**

LYMPHOCYTES from patients with malignant diseases can be differentiated from those of healthy donors or donors with non-malignant disorders on the basis of changes in the structuredness of cytoplasmic matrix (SCM) induced by cancer basic protein (CaBP), tumour-tissue associated antigens and phytohaemagglutinin (PHA) (Cercek, Cercek and Garrett, 1974*b*; Cercek, Cercek and Franklin, 1974*a*; Cercek and Cercek, 1975*a*, *b*). Changes in the SCM are measured on cell suspensions with the technique of fluorescence polarization in a fluorescence spectrophotometer of high sensitivity (Cercek, Cercek and Ockey, 1973). These measurements yield data on average changes in the SCM over whole cell populations, and cannot yield estimates of the percentages of cells which change their SCM on stimulation. We have, therefore, extended the technique of fluorescence polarization to measurements of changes in the SCM on single cells. This paper describes the SCM technique for single cells and presents data on SCM distributions and fractions of lymphocyte populations which respond to stimulations with CaBP and PHA in healthy donors and in patients with malignant disorders.

### MATERIALS AND METHODS

*Preparation of lymphocytes.*—Human lymphocytes were prepared from blood collected in Searle-LH/10 lithium heparin-containing vials. Lymphocytes in a pure state (>90%) were obtained by the Ficoll–Triosil gradient separation (Harris and Ukaejiofo, 1969). Details of these procedures were described before (Cercek *et al.*, 1974*b*). Lymphocytes were suspended in TC Medium 199 (Wellcome Ltd) at a concentration of approximately $6 \times 10^6$ cells/ml.

*Stimulation of lymphocytes.*—Aliquots of 0·5 ml of lymphocyte suspensions were incubated for 30 min at 37°C with either 0·05 ml of a 5 times diluted reagent-grade PHA (Wellcome Ltd) or 0·05 ml of partly purified CaBP solution. The concentration of CaBP was, on the basis of the dose response curve for the particular batch, adjusted to give a maximum response with respect to the number of lymphocytes. CaBP was a gift from Dr J. P. Dickinson, Cookridge Hospital, University Department of Radiotherapy, Leeds.

*Preparation of FDA solutions.*—Fluorescein diacetate (FDA) is practically insoluble in water. Aqueous solutions of FDA in complete phosphate buffered saline (PBS) (Paul, 1970) are, therefore, prepared by sequential dilution of a stock solution of 25 mg of recrystallized FDA/ml of Analar grade propanone or butan-2-on (Fluka Fluorochem Ltd). 0·02 ml of the FDA stock solution is first injected into 100 ml of sterile PBS. A slightly milky solution is obtained. This solution tends to flocculate and must, therefore, be immediately sub-diluted by injecting 10 ml of this solution into 100 ml of sterile PBS, with a further dilution by injecting 20 ml into 50 ml of sterile PBS. This latter solution (0·31 $\mu$M FDA in PBS) is used in SCM measurements. We have noticed that recent batches of the organic solvents and FDA contain impurities which decrease the SCM of lymphocytes. However, the above sequential dilution decreases the impurities below the threshold level at which they would start to affect the lymphocytes and thus abrogate mitogenic or antigenic stimulations.

*Measurements of SCM.*—The SCM of lymphocytes was measured with the technique of fluorescence polarization. The technique is based on the excitation of fluorescein molecules produced by enzymatic hydrolysis of the non-fluorescing substrate, fluorescein diacetate (FDA), in the cytoplasm of living cells with polarized light, and measurement of the degree of polarization of the emitted fluorescence. Three drops of control or of incubated lymphocyte suspensions (approximately $2 \times 10^5$ lymphocytes) were put on a pre-cleaned and fluorescence-free, select microslide (Chance Propper Ltd). The same volume of a 0·31 $\mu$M FDA solution in complete PBS solution was added, mixed with a sterile hypodermic needle and covered with a pre-cleaned and fluorescence-free glass coverslip No. 0 (Chance Propper Ltd). Under our experimental conditions we did not find it necessary to seal the cover slip to prevent loss of liquid by evaporation. After measuring about 50 cells a new slide was prepared. For lymphocytes stimulated with PHA or CaBP the incubation times of each new slide were approximately 30 min. For each histogram 150 to 200 cells were measured. The fluorescence polarization measurements of single cells were carried out at 25°C with a Zeiss microscope-fluorometer-01 equipped with an epi-fluorescence condenser III/RS, an electro-mechanically operated rotating polarizer (constructed for us by Zeiss) fitted between the photometer attachment and the tube body. To prevent photobiological effects during measurements on living cells and to obtain maximum excitation of fluorescein molecules, an interference filter transmitting light between 400 nm and 490 nm was installed in front of the excitation polarizer transmitting vertically polarized light. The epi-condensor was equipped with a reflector filter (cut-off below 500 nm) and a barrier filter (cut off below 505 nm). In all measurements the Zeiss Planapo 40/1·0 oil objective and Optovar 2·0 were used. The diameter of the measuring diaphragm in front of the photo-multiplier was selected to fit the size of the lymphocyte image. No corrections for scattered light or background fluorescence were necessary. The intensities of the emissions parallel, $I_{\parallel}$, and perpendicular, $I_{\perp}$, to the electric vector of the plane polarized exciting light beam were displayed on the digital read-out instrument. The fluorescence polarization values, $P$, were calculated from the relationship: $P = (I_{\parallel} - QI_{\perp})/(I_{\parallel} + QI_{\perp})$, where $Q$ denotes a correction factor for the unequal transmission of the two components of polarized light through the optical system. Under our experimental conditions $Q$ was 1·47. The factor $Q$ was derived from measurements of the intensities of the practically non-polarized fluorescence emitted from a 200 $\mu$M fluorescein solution in ethanol recorded with the emission polarizer in the parallel ($i_{\parallel}$) and perpendicular ($i_{\perp}$) position, *i.e.* $Q = i_{\parallel}/i_{\perp}$. The accuracy of this correction was checked by measuring the fluorescence polarization values, $P$, on a series of 50 $\mu$M fluorescein solutions in water–glycerol mixtures of varying viscosities at 25°C, first in the Perkin–Elmer fluorescence spectrophotometer MPF-4 and then under the microscope-fluorometer. The fluorescence polarization values, $P$, in the ranges of 0·150 to 0·280, differed by less than $\pm 2\%$.

*Calculation of fractions of lymphocyte population responding to PHA and CaBP.*—The minimum proportion ($F$) of lymphocytes which must undergo a change of $P$ in order to convert the control distribution into the treated distribution can be calculated as follows:

$$F = \tfrac{1}{2} \sum_{v} (F_{s,\,i} - F_{c,\,i})_{F_s > F_c} + \tfrac{1}{2} \sum_{v} (F_{c,\,i} - F_{s,\,i})_{F_c > F_s} \qquad \text{(I)}$$

where $F_{s,\,i}$ and $F_{c,\,i}$ denote fractions of lymphocytes per polarization interval of $P = 0{\cdot}02$ and the subscripts: $i$, the running interval; $s$, stimulated lymphocytes and $c$, control lymphocytes.

### RESULTS

The control SCM values of individual lymphocytes in populations from 5 healthy donors and 7 patients with cancer range from $P = 0{\cdot}10$ to $P = 0{\cdot}30$ with mean values of $P$ ranging from 0·188 to 0·221 (Table I). Examples of distributions of $P$ values within a population of lymphocytes from a healthy donor and a donor with cancer are shown as histograms in Fig. 1 and 2. The fluorescence intensity of cells with equal SCM values varied up to threefold. Repeat measurements of SCM values on the same cell differed by less than 2%. The mean SCM values, $P$, and fractions of lymphocyte populations, $F$, which respond with changes in the SCM to stimulations with PHA and CaBP are summarized in Table I. It can be seen that in the 5 healthy donors studied 45 to 54% of the lymphocyte population responds with a decrease in the SCM to PHA stimulation and only between 3 to 5% respond to CaBP stimulation. In the 7 cancer patients studied 36 to 45% of the lymphocyte population responded with a decrease in the SCM to CaBP stimulation and 15 to 23% to PHA stimulation.

Examples of the distributions of SCM values after stimulation with PHA and CaBP in lymphocytes from a healthy donor are shown in Fig. 1 and in those from a cancer patient in Fig. 2. The SCM values of stimulated lymphocytes range from $P = 0{\cdot}08$ to $P = 0{\cdot}240$.

### DISCUSSION

The present study shows that changes in the SCM can be measured on single cells using a microscope fluorometer under polarized light. The summary of the results on mean values of the SCM ($P$ in Table I) corroborates our previous findings on lymphocyte suspensions, measured in a fluorescence spectrophotometer, that changes in SCM induced by PHA and CaBP stimulation can be used to differentiate between lymphocytes from healthy donors and those from donors with malignant disease (Cercek and Cercek, 1975*a*; Cercek *et al.*, 1974*a*). The SCM values of individual lymphocytes in populations of unstimulated lymphocytes from healthy donors and from patients with cancer range from $P = 0{\cdot}10$ to $P = 0{\cdot}30$ (Fig. 1 and 2), with mean values of $P$ between 0·188 and 0·221 (Table I). This is in agreement with measurements on unstimulated lympho-

TABLE I.—*Mean SCM Values, P, and Fractions, F, of Lymphocyte Populations Responding to PHA and CaBP Stimulation*

| Diagnosis | | Age and sex | $P_{Control}$ | $P_{PHA}$ | $F_{PHA}$ | $P_{CaBP}$ | $F_{CaBP}$ |
|---|---|---|---|---|---|---|---|
| Healthy | | 22 M | 0·221 | 0·150 | 0·54 | 0·212 | 0·04 |
| | | 21 M | 0·220 | 0·149 | 0·53 | 0·214 | 0·05 |
| | | 28 M | 0·205 | 0·154 | 0·51 | 0·200 | 0·03 |
| | | 46 M | 0·215 | 0·158 | 0·48 | 0·206 | 0·04 |
| | | 45 F | 0·200 | 0·155 | 0·45 | 0·192 | 0·05 |
| Carcinoma of: | epiglottis | 63 M | 0·188 | 0·185 | 0·15 | 0·149 | 0·36 |
| | alveolus | 52 M | 0·210 | 0·205 | 0·19 | 0·155 | 0·45 |
| | lung | 54 M | 0·202 | 0·200 | 0·23 | 0·158 | 0·39 |
| | larynx | 62 M | 0·203 | 0·196 | 0·17 | 0·160 | 0·40 |
| | tongue | 83 F | 0·201 | 0·198 | 0·16 | 0·155 | 0·42 |
| | lung | 50 M | 0·214 | 0·216 | 0·20 | 0·178 | 0·43 |
| | lower lip | 64 M | 0·206 | 0·201 | 0·21 | 0·147 | 0·42 |

Standard errors of $P$ values are $< \pm\, 0{\cdot}003$.

Fig. 1.—Distribution of SCM values in a population of lymphocytes from a healthy donor before and after stimulation with PHA and CaBP.

Fig. 2.—Distribution of SCM values in a population of lymphocytes from a donor with cancer before and after stimulation with PHA and CaBP.

cyte suspensions carried out in a fluorescence spectrophotometer which also showed that there are no differences between the unstimulated SCM values of healthy donors and patients with cancer (Cercek *et al.*, 1974*a*). Furthermore, the mean values of SCM derived from single cell measurements (Table I) confirm that lymphocytes from cancer patients respond to stimulation by CaBP with a decrease in the SCM, but not to that by PHA, whereas lymphocytes from healthy donors respond to stimulation by PHA but not to that by CaBP. The population distribution of *P* values in stimulated lymphocytes is shifted to lower SCM values (Fig. 1 and 2). Since basic values of the SCM and responses to stimulations are not age-dependent (*loc. cit.*), in this study cancer patients and healthy donors are not age matched.

From the magnitude, mode and time dependence of the SCM response of lymphocytes to PHA and CaBP as measured on cell suspensions, we inferred that the same size and type of the population (T-cells?) may be involved (*loc. cit.*). The results in Table I show that indeed large fractions of lymphocyte populations respond to both PHA and CaBP. In healthy donors at least 45 to 54% of the lymphocyte population decreases the SCM on PHA stimulation, and in cancer patients at least 36 to 45% of the lymphocyte population responds to CaBP stimulation. Our previous results on lymphocyte suspensions using the fluorescence spectrophotometer technique suggested that cancer patients' lymphocytes do not change the mean SCM on PHA stimulation and similarly those from healthy donors do not on CaBP stimulation. The mean values of SCM (*P* in Table I) lead to the same conclusion. However, the estimates of *F* indicate that in healthy donors 3 to 5% of the lymphocyte population changes its SCM on CaBP stimulation and that in cancer patients 15 to 23% of the cell population responds to PHA. The magnitude of response of the responding fractions could, however, be relatively slight.

Figs. 1 and 2 provide no evidence of bimodality in the value of *P* in stimulated lymphocytes. They do not therefore support the idea that the lymphocytes fall into two classes, responders and non-responders, in spite of our large estimates of *F*, "the responding fraction".

It must be stressed that these estimates of *F* are minimal and are even compatible with a uniform shift downwards in the *P* values of all the lymphocytes.

The magnitude of the responses of single cells to stimulations by PHA and CaBP cannot be derived from the present results. To elucidate these points, further studies on SCM changes of single lymphocytes are in progress.

In conclusion, this study has demonstrated the feasibility of SCM measurements on single cells. It offers not only an alternative to measurements of the SCM on cell suspensions in a fluorescence spectrophotometer (Cercek *et al.*, 1973; 1974*a*, *b*), but also information on the SCM distributions in cell populations and changes in the SCM within the population on stimulation or treatment with different agents.

The authors thank the consultants of the Christie Hospital and Dr G. Read for blood samples from patients. This work was supported by grants from the Cancer Research Campaign and the Medical Research Council.

### REFERENCES

CERCEK, L. & CERCEK, B. (1975*a*) Changes in the Structuredness of Cytoplasmic Matrix of Lymphocytes as a Diagnostic and Prognostic Test for Cancer. In *Proceedings of the XI International Cancer Congress, Florence, 1974. Vol. 1, Cell Biology and Tumor Immunology.* Amsterdam: Excerpta Medica. p. 318.

CERCEK, L. & CERCEK, B. (1975*b*) Apparent Tumour Specificity with the SCM Test. *Br. J. Cancer*, **31**, 252.

CERCEK, L., CERCEK, B. & FRANKLIN, C. I. V. (1974*a*) Biophysical Differentiation between Lymphocytes from Healthy Donors, Patients with Malignant Diseases and other Disorders. *Br. J. Cancer*, **29**, 345.

CERCEK, L., CERCEK, B. & GARRETT, J. V. (1974*b*) Biophysical Differentiation between Normal Human and Chronic Lymphocytic Leukaemia Lymphocytes. In *Lymphocyte Recognition and Effector Mechanisms.* Ed. K. Lindahl-Kiessling and K. Osoba. New York, London: Academic Press. p. 553.

CERCEK, L., CERCEK, B. & OCKEY, C. H. (1973) Structuredness of the Cytoplasmic Matrix and Michaelis-Menten Constants for the Hydrolysis of FDA during the Cell Cycle in Chinese Hamster Ovary Cells. *Biophysik*, **10**, 187.

HARRIS, R. & UKAEJIOFO, E. O. (1969) Rapid Preparation of Lymphocytes for Tissue-Typing. *Lancet*, ii, 327.

PAUL, J. (1970) *Cell and Tissue Culture.* Edinburgh and London: E. and S. Livingstone. p. 91.